# The First Estimates of Marbled Cat *Pardofelis marmorata* Population Density from Bornean Primary and Selectively Logged Forest

**DOI:** 10.1371/journal.pone.0151046

**Published:** 2016-03-23

**Authors:** Andrew J. Hearn, Joanna Ross, Henry Bernard, Soffian Abu Bakar, Luke T. B. Hunter, David W. Macdonald

**Affiliations:** 1 Wildlife Conservation Research Unit (WildCRU), Department of Zoology, University of Oxford, Oxford, United Kingdom; 2 Institute for Tropical Biology and Conservation, Universiti Malaysia Sabah, Kota Kinabalu, Sabah, Malaysia; 3 Sabah Wildlife Department, Kota Kinabalu, Sabah, Malaysia; 4 Panthera, New York, New York, United States of America; Texas A&M University, UNITED STATES

## Abstract

The marbled cat *Pardofelis marmorata* is a poorly known wild cat that has a broad distribution across much of the Indomalayan ecorealm. This felid is thought to exist at low population densities throughout its range, yet no estimates of its abundance exist, hampering assessment of its conservation status. To investigate the distribution and abundance of marbled cats we conducted intensive, felid-focused camera trap surveys of eight forest areas and two oil palm plantations in Sabah, Malaysian Borneo. Study sites were broadly representative of the range of habitat types and the gradient of anthropogenic disturbance and fragmentation present in contemporary Sabah. We recorded marbled cats from all forest study areas apart from a small, relatively isolated forest patch, although photographic detection frequency varied greatly between areas. No marbled cats were recorded within the plantations, but a single individual was recorded walking along the forest/plantation boundary. We collected sufficient numbers of marbled cat photographic captures at three study areas to permit density estimation based on spatially explicit capture-recapture analyses. Estimates of population density from the primary, lowland Danum Valley Conservation Area and primary upland, Tawau Hills Park, were 19.57 (SD: 8.36) and 7.10 (SD: 1.90) individuals per 100 km^2^, respectively, and the selectively logged, lowland Tabin Wildlife Reserve yielded an estimated density of 10.45 (SD: 3.38) individuals per 100 km^2^. The low detection frequencies recorded in our other survey sites and from published studies elsewhere in its range, and the absence of previous density estimates for this felid suggest that our density estimates may be from the higher end of their abundance spectrum. We provide recommendations for future marbled cat survey approaches.

## Introduction

The marbled cat *Pardofelis marmorata* is a small, elusive, forest-dependent felid whose wide distribution spans the Indomalayan ecorealm, from Eastern India and Nepal, to Yunnan province, China, and throughout mainland Southeast Asia to the islands of Sumatra and Borneo [1]. This little known wild cat possesses a uniquely marbled coat pattern, from which its name is derived, and a distinctly thick and disproportionately long tail, which is characteristically held in a horizontal fashion when walking. The tail provides a useful counterbalance when climbing, and is likely an adaptation for a particularly arboreal lifestyle [2], although, as with much of this species’ natural history, this is unconfirmed. In captivity the marbled cat is an adept climber [3], and in the wild it has been observed descending, head-first, down the trunk of a large tree, an ability only previously reported in clouded leopards *Neofelis* spp. and Margays *Leopardus wiedi* [4]. The marbled cat’s diet remains unknown [5], but arboreal prey are assumed to be important and there is an observation of an individual stalking birds in the canopy [6] and another potentially preying on a juvenile Phayre’s leaf monkey *Trachypithecus phayrei* [7]. Nevertheless, despite their obvious arboreal adaptations the scientific literature includes camera trapping records of marbled cats walking on the ground [8–22], so this felid’s activities are clearly not restricted to the trees.

The marbled cat is widely considered to be a rare felid, whose populations are thought to be declining [1,2,23], yet there are no estimates of its abundance in any part of its range [24], hampering robust assessment of its conservation status [1]. Camera trap surveys undertaken within marbled cat range typically yield very few photographic captures [9–22]. Such low capture success has likely hitherto precluded efforts to estimate this felid’s population density through capture-recapture analyses. Whether these low capture rates are a result of true rarity, or a reflection of the species’ semi-arboreal nature or habitat use is unclear.

While knowledge of the marbled cat’s status remains shrouded in uncertainty, it is clear that the loss of forest across their range continues at an ever increasing rate [25,26] and that indiscriminate poaching of these cats continues unabated [15,27,28], presenting a significant potential threat [1]. As such, there is an increasing need to derive scientifically robust, range-wide estimates of the status of the marbled cat to facilitate the development of appropriate conservation measures. Here we use data stemming from intensive, felid-focused camera trapping surveys of a range of habitat types in Sabah, Malaysian Borneo to investigate the distribution and abundance of marbled cats. We produce the first marbled cat population density estimates from both primary and selectively logged forest areas using spatially-explicit capture-recapture modelling within a Bayesian framework.

## Materials and Methods

### Ethics Statement

The Economic Planning Unit of Malaysia, Sabah Biodiversity Council, Sabah Parks, Sabah Forestry Department, Sabah Wildlife Department and Yayasan Sabah reviewed all sampling procedures and approved permits for the work conducted. We applied non-invasive methods for data gathering and hence approval from an Institutional Animal Care and Use Committee or equivalent animal ethics committee was not required.

### Study Areas

**Fig 1:**
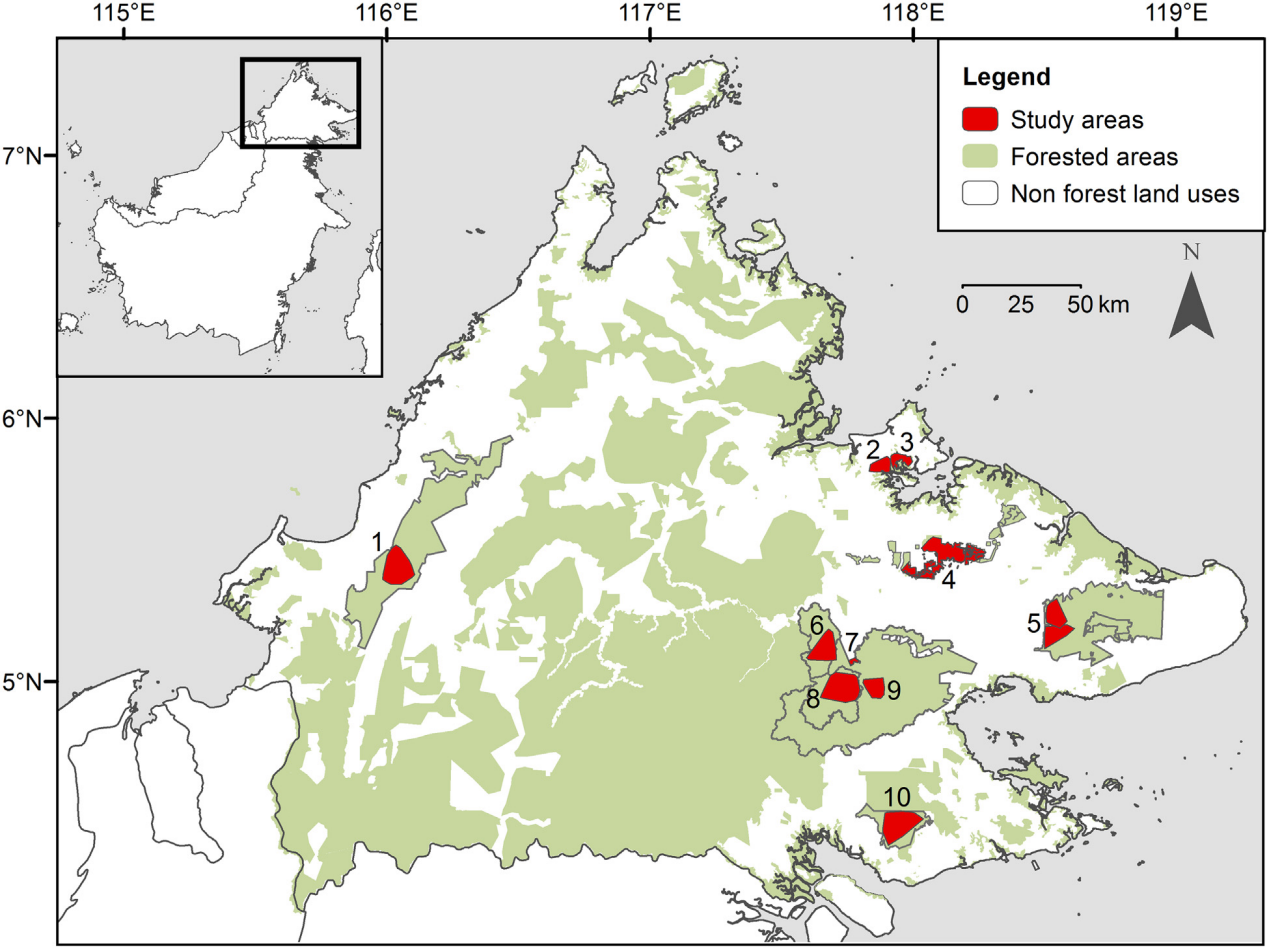
Locations of the eight forest and two oil palm plantation survey areas for marbled cats in Sabah, Malaysian Borneo. Inset shows the island of Borneo, and the main map shows the Malaysian state of Sabah. Numbered polygons represent the different study areas: 1. Crocker Range Park; 2. Minat Teguh plantation; 3. Kabili-Sepilok Forest Reserve; 4. Lower Kinabatangan Wildlife Sanctuary; 5. Tabin Wildlife Reserve (North and South); 6. Malua Forest Reserve; 7. Danum Palm plantation; 8. Danum Valley Conservation Area; 9. Ulu Segama Forest Reserve; 10. Tawau Hills Park. Density estimation using SECR analysis was possible in three of these areas: Danum Valley, Tabin and Tawau.

We systematically surveyed eight forest areas and two oil palm plantations in Sabah, Malaysian Borneo with camera traps between May 2007 and December 2013 (Fig 1). The survey areas were broadly representative of the range of habitat types and the gradient of anthropogenic disturbance and fragmentation present in contemporary Sabah [29] (Table 1). Survey areas included three primary forests: Danum Valley Conservation Area (Danum Valley); Tawau Hills Park (Tawau) and Crocker Range Park (Crocker), which range in elevation from lowland and hill dipterocarp to montane forest. We surveyed five selectively logged areas: Lower Kinabatangan Wildlife Sanctuary (Kinabatangan), Tabin Wildlife Reserve (sub-divided into two areas, Tabin North and South, see below), and Kabili-Sepilok, Malua and Ulu Segama Forest Reserves, which vary both in the degree of logging disturbance they were exposed to and in their levels of isolation and fragmentation. We also surveyed two oil palm plantations: Danum Palm and Minat Teguh plantations, which were both contiguous with areas of forest.

**Table 1:** Details of the eight forest and two oil palm plantation study areas in Sabah, Malaysian Borneo.

Study area	FMU size (km^2^)	Location (Lat/ Lon)	Dominant landcover type(s)	Level of fragmentation
Crocker	1399	5° 26’ N, 116° 02’ E	Primary, hill dipterocarp, sub-montane & montane.	Large, relatively isolated forest block.
Danum Valley	438	4° 58’ N, 117° 46’ E	Primary, lowland & hill dipterocarp.	Part of ca. 1 million ha Central Sabah Forest complex [29].
Kabili-Sepilok	42.9	5° 51’ N, 117° 57’ E	Partially selectively logged, lowland dipterocarp, heath forest & mangrove.	Small, isolated fragment. Possible connectivity along coastal mangrove system
Kinabatangan	260	5° 29’ N, 118° 08’ E	Selectively logged, mosaic of forest types, including riparian forest, seasonally flooded forest, swamp forest, limestone forest.	Highly fragmented. Contiguous with 250 km^2^ state owned Forest Reserves and privately owned forest patches.
Malua	340	5° 08’ N, 117° 40’ E	Twice-logged (1960s & 2006–2007), lowland dipterocarp. High density of open logging roads and skid trails.	Part of ca. 1 million ha Central Sabah Forest complex [29].
Tabin (North and South)	1,205	5° 14’ N, 118° 51’ E	Selectively logged (1969–1989), lowland dipterocarp. Low density of open and semi-closed logging roads.	Large, relatively isolated forest block. Possible connectivity with coastal mangrove to north.
Tawau	280	4° 27’ N, 117° 57’ E	Primary, lowland & hill dipterocarp, sub-montane & montane.	Large, relatively isolated forest block, contiguous with commercial Forest Reserve to north.
Ulu Segama	2029	4° 59’ N, 117° 52’ E	Selectively logged (1978–1991), lowland dipterocarp. Rehabilitation planting ongoing [30]. Medium density of open and semi-closed logging roads.	Part of ca. 1 million ha Central Sabah Forest complex [29].
Danum Palm	NA	5° 05’ N, 117° 46’ E	Semi-mature (planted in 2000), terraced oil palm plantation. Largely open understorey. Semi-natural scrub bordering one large river and one stream.	Shares eastern and western borders with ca. 1 million ha Central Sabah Forest complex [29].
Minat Teguh	NA	5° 50’ N, 117° 53’ E	Mature (planted in 1995), oil palm plantation. Largely open understorey. Border fringed with mangrove.	Shares eastern border with Kabili-Sepilok Forest Reserve

FMU: Forest Management Unit.

### Methods

We used passive infrared digital camera traps of varying models: Bushnell Trophycam 2010 (Bushnell Corporation, KS, USA), Cuddeback Capture (Non Typical Inc., WI, USA), Panthera V3 (Panthera, NY, USA), Reconyx HC500 and PC800 (Reconyx Inc., WI, USA) and Snapshot Sniper P41 (Snapshot Sniper LLC, OK, USA). Due to varying equipment and logistical constraints, camera trap grids differed in size and effort, and cameras were deployed according to one of two protocols, (i) Split-grid, where the entire grid is sequentially surveyed in two halves and (ii) Simultaneous, where all camera stations are deployed in a single phase (Table 2). Camera stations were un-baited and separated by approximately 1.5–2.0 km. In our forest surveys we preferentially deployed camera stations along established human trails, newly cut trails and ridgelines. In the absence of an existing human trail we attempted to recreate an established trail by clearing an approximately 0.6 m wide section of trail free of dense vegetation, woody saplings and leaf litter, for approximately 50 m either side of the camera station. Established trails were also cleared in this fashion in the vicinity of the camera station, particularly those that were not well used. Where available, camera stations were also situated along old, unsealed logging roads. Such roads formed the majority of camera stations in two of the selectively logged sites (Malua and Ulu Segama), but formed a small proportion of camera stations or were absent in the other five forest sites (Table 2). For the plantation surveys we deployed cameras along access roads, human paths and narrow stretches of terrace (Danum Palm only). In all survey areas, cameras were positioned around 40–50 cm above the ground and arranged in pairs to enable both flanks of the animal to be photographed simultaneously, to permit subsequent identification of individuals based on their unique pelage patternation.

**Table 2:** Camera trap survey specifications and marbled cat photographic capture data derived from intensive camera trap surveys of multiple study areas in Sabah, Malaysian Borneo.

Study area	Camera trap grid					Survey effort and marbled cat capture data						
	Area (km^2^)^a^	Protocol ^b^	No. cam. stations	No. cam. stations on road / trail	Mean elevation and range (m.a.s.l)	Survey Dates	No. trap days	No. independent captures^c^		Detection frequency^d^	No. different animals^e^	
								Adults	cubs		Adults	cubs
Crocker	149.7	Sim.	35	3 / 32	1029 (383–1452)	6/10/11–27/2/12	4059	11	0	0.27	5 (3)	0
Danum Valley^f^	157.0	Split-grid	79	0 / 79	384 (153–804)	24/3/12–6/10/12	5837	39	0	0.67	17 (10)	0
Kabili Sepilok	49.4	Sim.	35	0 / 35	66 (8–134)	9/2/11–25/5/11	2054	0	0	0	0	0
Kinabatangan	359.5	Split-grid	66	0 / 66	35 (5–135)	24/7/10–17/12/10	4340	5	0	0.12	3 (2)	0
Malua	102.8	Sim.	38	38 / 0	177 (68–286)	9/7/08–12/2/09	3869	5	0	0.13	3 (2)	0
Tabin North^f^	71.4	Sim.	37	1 / 36	140 (11–407)	16/12/09–22/4/10	3300	27	1	0.82	8 (2)	1
Tabin South	72.9	Sim.	37	11 / 27	209 (62–431)	18/9/09–11/1/10	3162	15	0	0.47	4 (1)	0
Tawau^f^	149.0	Sim.	77	0 / 77	706 (209–1195)	21/10/12–30/12/13	17397	72	1	0.41	28 (4)	1
Ulu Segama	60.1	Sim.	22	19 / 3	252 (150–408)	24/5/07–18/10/07	2847	7	1	0.25	5 (2)	1
Danum Palm	7.8	Sim.	23	NA	210 (120–295)	15/3/09–7/7/09	2212	5	0	0.23	1 (0)	0
Minat Teguh	44.0	Sim.	33	NA	23 (1–49)	26/5/11–18/8/11	1960	0	0	0	0	0

^a^ Camera trap grid area is defined by a 100% Minimum Convex Polygon around all camera stations.

^b^ We followed two survey protocols, Split-grid: where the entire grid was sequentially surveyed in two halves, and Sim.: Simultaneous, where all camera stations were deployed in a single phase.

^c^ Number of photographic captures of different individuals or images obtained more than 1 hour apart.

^d^ The number of independent adult photographic captures per 100 trap days.

^e^ Values within parentheses represent the number of independent photographic captures that did not permit identification to individual.

^f^ SECR density estimation was possible at these sites.

Where resulting photographic capture data from each site permitted, we estimated population densities of marbled cats using a Spatially Explicit Capture Recapture (SECR) model undertaken within a Bayesian framework [31], implemented in the R (version 3.1.2 [32]) package SPACECAP (version 1.1.0 [33]). This approach incorporates a model of individual movements with one that describes detection by camera traps [34,35]. For each study site we compiled the number of photographs of each individual at each camera station and developed a capture history for each identified animal. Identification of animals was independently undertaken by a minimum of two people. We were unable to reliably distinguish the sex of marbled cats from all photographs, and so both sexes were analysed together. Finer sampling interval lengths may improve precision of density estimates in SECR analyses [36] and so we considered each 24-hour period as a sampling occasion. We limited our sampling duration to approximately 4 months (100–120 days, Table 2), which is a duration applied in similar studies to approximate population closure (e.g., [37,38]). Surveys typically included lengthy camera set-up, transition (Split-grid protocol only) and collection phases, thus the total survey duration of each study site exceeded these closed periods. As a consequence, we selected closed survey periods during which camera trap effort, and, in turn, marbled cat photographic capture rates were maximised. Due to logistical constraints during our Tabin survey, which followed a Split-grid protocol, the transition phase in Tabin exceeded 50 days, and so we present these two sub-areas as two distinct surveys: Tabin North and South.

We generated potential home range centres by delineating a grid of regularly spaced points, with a mesh size of 0.16 km^2^, within a polygon defined by the addition of a buffer to the outermost coordinates of the three trapping grids. This is known as the state space. We systematically increased buffer size during a sequence of preliminary runs until detection probability at the edge of the state space was negligible; we deemed a buffer size of 10 km sufficient for all sites. We classified each potential home range centre as either habitat or unsuitable-habitat using a GIS (ArcMap 10.2, ESRI, Redlands, California, USA) in conjunction with habitat data derived from field knowledge and hi-resolution aerial images from Google Earth (DigitalGlobe). Marbled cats are thought to be forest dependent and not found in oil palm plantations [1], and so we considered forested areas (both pristine and disturbed) as habitat and all other non-forest land uses, as unsuitable. For all analyses SPACECAP was run using a half normal model, with 100,000 iterations, a burn-in of 15,000 and a thinning rate of 1. We set data augmentation to 180, 800 and 140 for our Tabin, Danum Valley and Tawau analyses, respectively, following a series of preliminary runs, increasing data augmentation where necessary to ensure that ψ, the ratio of the estimated abundance within the state space to the maximum allowable number defined by the augmented value, did not exceed 0.8. We assessed model parameter convergence by means of Geweke tests; z scores falling between -1.64 and 1.64 were deemed acceptable.

## Results

We recorded marbled cats in all forest study sites apart from Kabili-Sepilok, although photographic capture success varied greatly between areas (Table 2). We only obtained sufficiently high marbled cat detection frequencies to permit density estimation from Danum Valley, Tabin North and Tawau (Table 3). We recorded a single cub, on one occasion, in each of Tabin North, Tawau and Ulu Segama. We did not detect any marbled cats in Minat Teguh but an individual marbled cat was recorded on five occasions at a single camera station in Danum Palm, which was located at the very border of the plantation/interface with the Ulu Segama Forest Reserve.

**Table 3:** Sampling specifications and marbled cat capture data from the closed survey periods in Danum Valley, Tabin North and Tawau.

Study area	Closed survey period	No. sampling occasions	No. trap days	No. captures^a^		No. different animals		No. captures per individual^b^
				Adults	cubs	Adults	cubs	
Danum Valley	25/05/2012–21/09/2012	120	4319	22 (4)	0	15	0	5(2), 2(2), 2(1), 2(1), 1(1), 1(1), 1(1), 1(1), 1(1), 1(1), 1(1), 1(1), 1(1), 1(1), 1(1)
Tabin North	11/01/2010–20/04/2010	100	2815	25 (3)	1	8	1	8(1), 6(1), 5(2), 2(2), 1(1), 1(1), 1(1), 1(1)
Tawau	14/12/2012–12/04/2013	120	6641	35 (1)	0	15	0	8(5), 6(4), 4(3), 4(2), 2(2), 2(1), 1(1), 1(1), 1(1), 1(1), 1(1), 1(1), 1(1), 1(1), 1(1)

^a^ Number of independent photographic captures that were used in the SECR analysis. Values in parentheses represent the number of independent captures that were obtained within the closed period but did not permit individual identification and so were excluded from the analysis.

^b^ Values in parentheses represent the number of different camera stations that each individual was recorded at during the closed survey period.

Posterior SECR summaries of the model parameters from our three study sites that provided sufficient data for density estimation are provided in Table 4. The mean estimated marbled cat densities for Tabin North, Danum Valley and Tawau were 10.45 (SD: 3.38), 19.57 (SD: 8.35), and 7.10 (SD: 1.90) individuals per 100 km^2^, respectively, with 95% intervals of 4.01–17.37, 6.87–36.65 and 3.49–10.73 individuals per 100 km^2^, respectively. Bayesian *p*-values of our SECR models indicated that the models were of an adequate fit, and Geweke tests indicated that all model parameters converged. The movement parameters were similar for both Tabin and Danum Valley, but were substantially larger in Tawau, indicating that the home ranges of animals in that population are likely larger.

**Table 4:** Posterior summaries from the Bayesian-SECR model parameters of camera trap data from Danum Valley, Tabin North and Tawau.

Parameter	Danum Valley		Tabin North		Tawau	
	Mean (SD)	95% Lower—Upper HPD	Mean (SD)	95% Lower—Upper HPD	Mean (SD)	95% Lower—Upper HPD
σ	764 (215)	432–1155	643 (97)	470–832	2619 (511)	1777–3615
*λ*_*0*_	0.009 (0.006)	0.002–0.02	0.036 (0.014)	0.013–0.065	0.002 (0.001)	0.001–0.004
*ψ*	0.284 (0.121)	0.097–0.533	0.275 (0.093)	0.107–0.457	0.267 (0.078)	0.127–0.423
*N*	230.7 (98.5)	81–432	51.3 (16.6)	21–83	41 (11)	22–62
*D*	19.57 (8.35)	6.87–36.65	10.45 (3.38)	4.28–16.91	7.1 (1.9)	3.81–10.73
*p*-value	0.753		0.690		0.702	

σ: movement parameter, related to home range radius; λ_0_: baseline trap encounter rate, the detection parameter of the spatial explicit capture-recapture model; ψ: the ratio of the estimated abundance within the state space to the maximum allowable number defined by the augmented value; *N*: number of individuals in the state space; *D*: density (individuals per 100 km^2^).

Despite legislation prohibiting any hunting activity we found spent shotgun cartridges in seven of the forests we surveyed, Danum Valley being the only exception. We made no effort systematically to quantify poaching intensity but this is indicative that illegal poaching activities are widespread. No evidence of direct poaching of marbled cats was found.

## Discussion

We present the first published density estimates for the marbled cat from any part of its range. Typical of camera surveys of cryptic forest felids, our recapture rates were relatively low for many individuals, particularly in Danum Valley and Tabin North, limiting our ability to derive estimates of movement parameters, and so our estimates of density at these sites may be high. Our highest estimate of density was the primary, lowland hill dipterocarp forest of Danum Valley Conservation Area, which was approximately two times greater than that of both the lowland, selectively logged Tabin Wildlife Reserve and the primary, uplands of Tawau Hills Park. As there is both a considerable overlap in our 95% intervals and a lack of replicates our ability to make robust conclusions about the possible influence of habitat type and anthropogenic disturbance on marbled cat densities is limited. Nevertheless, our study provides tentative evidence that undisturbed, lowland hill forest may support higher densities than both disturbed lowland and undisturbed higher elevation forests in northern Borneo.

No estimates of marbled cat density are available to compare against those derived from the current study. Our marbled cat density estimates were higher than that of the Sunda clouded leopard *Neofelis diardi* in central Sabah (0.8–1.9 individuals per 100 km^2^), which were obtained using a SECR approach [38,39], but similar to estimates of leopard cat *Prionailurus bengalensis* density from the same area (9.6–16.5 individuals per 100 km^2^), which were also derived using an SECR approach [40]. The latter finding is unexpected given the presumed high abundance and rarity of the leopard cat and marbled cat, respectively, although such assumptions may be based on the leopard cat’s close association with logging roads leading to high observer encounter rates [40] and the marbled cat’s avoidance of such features. Indeed, McCarthy et al., [21] used camera traps to survey the Bukit Barisan Selatan National Park, Sumatra, and found that marbled cat occupancy peaked at moderate distances from roads (sealed and un-sealed).

Our density estimates may not be representative of other areas, and they may all stem from high density populations. Indeed, the relatively high capture frequencies of marbled cats in Danum Valley, Tabin North and Tawau, were approximately 2 to 6 times greater than that recorded in our other study sites and in previous intensive camera trap surveys elsewhere [9–22]. Although we found evidence of poaching activities in Tabin and Tawau, all these sites are protected areas with limited access. Thus, poaching intensity is likely to be low relative to other sites in Sabah, and indeed elsewhere in the marbled cat’s range. While we acknowledge the methodological limitations of comparing such indices of abundance across survey sites [41] the populations we surveyed in northern Borneo are likely to be from the higher end of their abundance spectrum.

The records of marbled cat presence in our four selectively logged survey sites, two of which provide confirmation of breeding activity, adds to the growing body of evidence that the species is able to tolerate some degree of habitat disturbance, e.g., [13], and highlights the potential conservation values of these forests to this felid. Our surveys of Kinabatangan, Tabin and Ulu Segama took place approximately 16–20 years post-harvest disturbance, and so these forests would have undergone substantial regrowth and recovery by this time. This is particularly true of Ulu Segama, where there has been intensive rehabilitation planting [29,30]. However, we also detected marbled cats in Malua, a particularly disturbed forest, less than one year post harvest. We recorded few marbled cats in the highly fragmented Kinabatangan, although we were unable to disentangle the potential interplay between forest disturbance, poaching intensity and fragmentation, from that of habitat association. While the absence of marbled cat captures from Kabili-Sepilok cannot be used to infer that the species has been extirpated, this forest patch may be too small and/or isolated for a population to persist. We did not detect any marbled cat activity within either plantation area, but we did record one individual walking along the forest/plantation interface in Danum Palm. While our plantation surveys were of limited scope, our data tend to support the view that palm oil plantations are rarely used by marbled cats [1], although the periphery may be utilised.

Indiscriminate hunting and poaching appears to be increasing region-wide, particularly in Lao PDR and Vietnam, where trade-driven intensive snaring is likely impacting wild felids, including the marbled cat [15,27,28]. It is becoming increasing important to gauge the status and monitor populations of threatened, non-*Panthera* felids, which currently lack such programmes and are rarely the focus of conservation effort and funding [27]. Although generating estimates of marbled cat density poses a significant challenge, we show that well designed surveys using well established techniques produce rigorous data useful for such. Of those surveys that yielded sufficient capture rates to permit density estimation, camera stations were primarily situated along existing and newly created human trails, and included very few stations along logging roads. It is possible that this contributed to our ability to detect this felid and so the efficacy of future marbled cat focused surveys may be improved by maximising off-road camera deployment. There are a number of equally intensive camera trapping studies now being undertaken within marbled cat range, most targeting the estimation of tiger *Panthera tigris* and clouded leopard population density. As these data become available further estimates of the density of marbled cat will better underpin ecological understanding and conservation planning of one of Asia’s most widely distributed, yet particularly elusive wild cats.

## Supporting Information

S1 Dataset SPACECAP input files for the three surveys areas which permitted SECR analysis. (XLSX)
